# A nationwide epidemiological and geodemographic analysis of lymphatic filariasis in Ecuador: a neglected and often forgotten disease in Ecuador

**DOI:** 10.3389/fpubh.2023.1270015

**Published:** 2023-11-14

**Authors:** Juan S. Izquierdo-Condoy, Patricio Naranjo-Lara, Jorge Vásconez-Gonzalez, Raul Fernandez-Naranjo, Romina Placencia-André, María G. Davila, Sarah J. Carrington, Esteban Ortiz-Prado

**Affiliations:** ^1^One Health Global Research Group, Universidad de las Américas, Quito, Ecuador; ^2^Pontificia Universidad Católica del Ecuador, Tecnologías PUCE TEC, Quito, Ecuador; ^3^Lugar, Medio y Sociedad, Universidad de las Américas, Quito, Ecuador

**Keywords:** lymphatic filariasis, vector-borne disease, mosquitoes, high altitude, *Culex*, South America

## Abstract

**Introduction:**

Lymphatic filariasis (LF) is a neglected parasitic disease transmitted by mosquitoes and affecting the lymphatic system. The aim of this study was to analyze the epidemiological and sociodemographic characteristics of patients with LF during the last 11 years of available data in Ecuador.

**Methods:**

A 11-year nationwide analysis of hospital admission and in-hospital mortality based on the National Institute of Statistics and Census (INEC) data was conducted in Ecuador from 2011 to 2021. The International Classification of Diseases 10th Revision (ICD-10) code for filariasis (ICD: B74) was used to retrieve information on severe LF as a proxy for incidence among 221 Ecuadorian cities.

**Results:**

A total of 26 hospital admissions and 3 deaths due to LF were registered. The highest mortality rate was found in populations over 80 years. Men accounted for 62.5% (*n* = 17) of total number of cases with an average incidence rate of 1.7 cases per/1,000,000, while females accounted for 34.6% (*n* = 9), representing 1 case per/1,000,000 woman. Cities located at lower altitude (459/1,000,000) reported higher incidence rates than those located at higher altitudes (7.4/1,000,000).

**Conclusion:**

This is the first study on LF in Ecuador. Although, Ecuador is not considered endemic for LF, we found evidence of the presence of this disease in recent years. The implementation and improvement of an adequate integrated epidemiological surveillance system will allow early identification of cases and therefore their respective treatment.

## 1. Introduction

Forgotten or Neglected Infectious Diseases are a group of infectious illnesses that mainly affect the poorest populations, often around the equator and in the southern hemisphere (1). These diseases are often seen among populations with limited access to health services, especially those living in tropical and remote rural areas worldwide (2). Within this group of emerging or re-emerging diseases, we have those transmitted by living organisms (vector-borne diseases), those transmitted by direct exposure to recreational or drinking water (water-borne diseases) or food contaminated by disease-causing microbes or pathogens (food-borne diseases) (3, 4).

Among the more than 20 neglected tropical diseases, LF stands out with over 800 million cumulative cases globally, endemic in at least 72 countries despite the World Health Organization’s commitment to its elimination (5). In 2018, approximately 51 million people were diagnosed with LF, primarily in low and middle-income countries (6). LF is prevalent in regions like Africa, Southeast Asia, the Caribbean, South America, and the Middle East. Notably, the WHO’s Global Program to Eliminate LF, launched in 2000, has contributed to a significant 74% reduction in LF cases by 2018, although the global burden remains concentrated mainly in Southeast Asia (2, 7).

Within the Americas, more than 12.5 million people are assumed to be at risk of this infection, most of them from Haiti and Brazil (8, 9). While filariasis is endemic in Brazil, Haiti, Guyana and the Dominican Republic, other countries in the region suffer from this disease, which almost always goes unnoticed (8). Most cases of LF worldwide are caused by the presence of the filarial nematode called *Wuchereria bancrofti*, followed by the *Brugia malayi* and *Brugia timori* species (10, 11). This parasitic vector-borne disease is transmitted by the bite of mosquitoes of the genus *Anopheles*, *Aedes*, *Culex* or *Mansoni*, most of them widely distributed between the Tropic of Capricorn and the Tropic of Cancer (10, 12, 13).

LF has been considered a potentially eradicable disease since no animal reservoirs for *W. bancrofti* have been described, and there are effective interventions to interrupt its transmission, as well as accurate diagnostic tools (12). The infection is usually contracted in childhood and causes no obvious damage (13). The morbidity of LF is expressed in the form of painful and profoundly disfiguring chronic manifestations that include lymphedema (acute dermatolymphangioadenitis), which can be accompanied by complications such as bacterial superinfection, male urogenital diseases (hydrocele and superficial scrotal lymphangiomatosis), breast lymphedema, inflammation of the vulva, and rheumatic and respiratory problems. These patients not only face physical limitations, but also face mental, social, and economic costs, which is why the WHO has classified this parasitic infection as the second most common cause of long-term disability after mental illness (14).

Long-term infection can lead to impairment of the lymphatic system, characterized by severe swelling of the extremities (lymphedema) and, subsequently, elephantiasis or lymphedema of the scrotum (hydrocele) (6). The mortality associated with filariasis is low, but the socio-health consequences of its chronic manifestations are remarkable (15).

**Figure 1:**
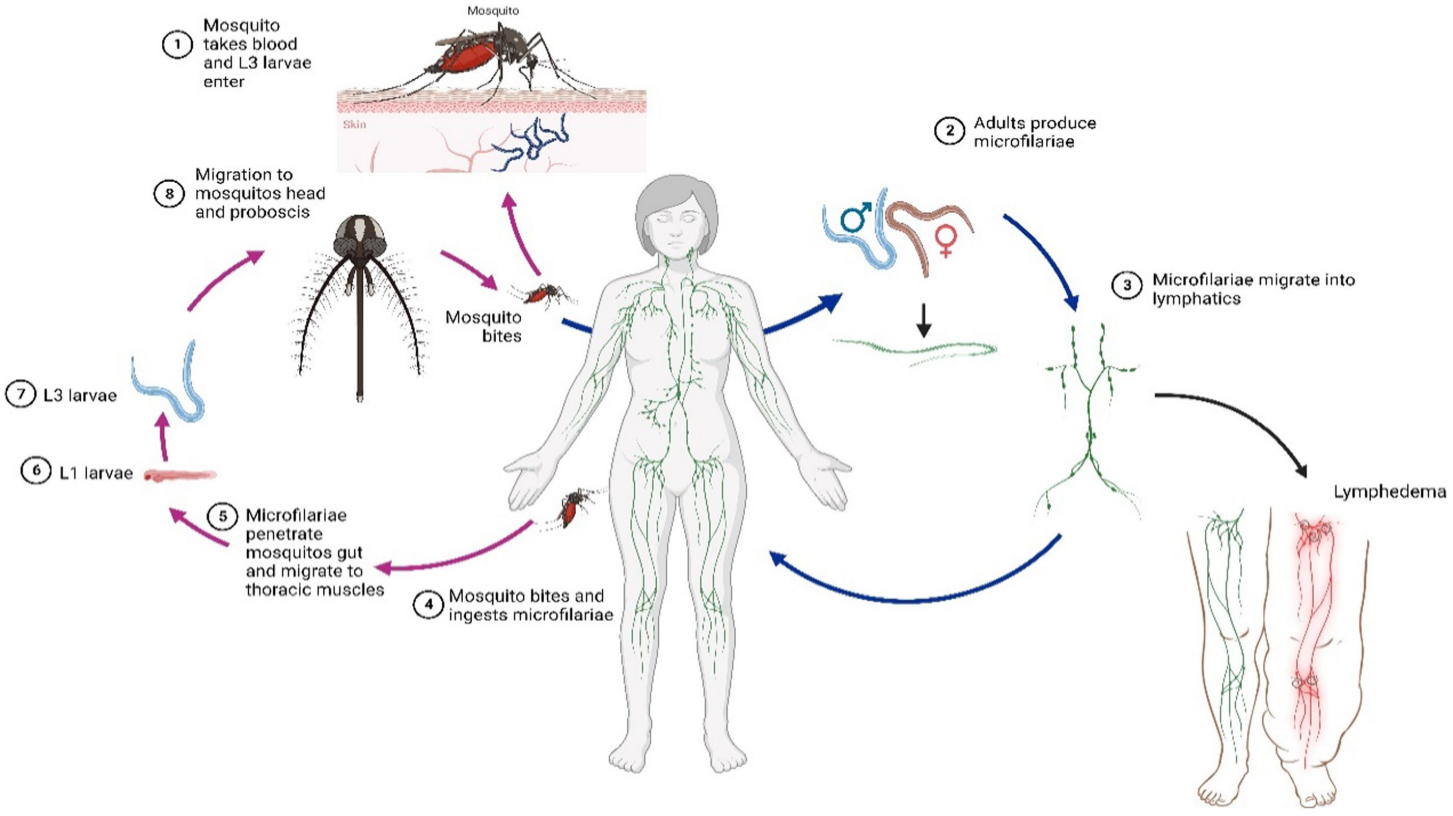
Filariasis internal and external life cycle.

The infection cycle starts when a mosquito ingests microfilariae, which develop into infective larvae (L3) in the mosquito’s thoracic muscles. These L3 larvae move to the mosquito’s proboscis and are transmitted to humans through bites. Upon entering the human host, L3 larvae molt into adult parasites (L4) within 9–10 days. These adults reside in the lymphatic system, where they mate and produce new microfilariae that circulate in the bloodstream (Figure 1).

By the year 2030, the WHO aims to achieve the elimination of LF, a goal that was set for the year 2020 (13). Although significant progress has been made in Latin America and the Caribbean, the disease burden is still underestimated and remains a cause for concern (16). Cases of filariasis due to *Mansonella* have been reported in the Amazon regions of Colombia and Peru, some very close to the border with Ecuador; however, there are no official data on the prevalence of LF in these countries (17, 18).

In Ecuador, no information on the prevalence of filariasis is available, and no current reports have been published despite our geographical diversity and vicinity with other countries where filariasis is endemic. In this context, our objective is to describe the epidemiological and sociodemographic characteristics of those patients with an official diagnosis of LF in Ecuador from 2011 and 2021 in Ecuador.

## 2. Methods

### 2.1. Study design

We conducted a cross-sectional and retrospective study to determine the patterns of filariasis morbidity, mortality, and spatial distribution using official hospital records for the period 2011 to 2021 (11 years) registered by National Institute of Census and Statistics (INEC) (19). This study was a country-wide population-based analysis of the epidemiology of ICD-10 codes cases in Ecuador.

Data from all patients with a hospital discharge diagnosis of (1) B740 (Filariasis due to *W. bancrofti*); (2) B741 (Filariasis due to *B. malayi*); (3) B742 (Filariasis due to *B. timori*); (4) B743 (Loiasis); (5) B744 (Mansonelliasis); (6) B748 (Other filariases); and (7) B749 (Filariasis, unspecified) was collected from each public and private health center accredited in Ecuador. Data gathered included patients’ morbidity, mortality, and case-fatality rates.

### 2.2. Sample and setting

Ecuador with an area of more than 283,560 km^2^ is the smallest country in the Andean mountainous region in South America. The country is divided into four geographical regions, the coast, the highlands, the Amazon region, and the Galapagos Islands (20).

Ecuador has different climates in each of its regions: the Western Pacific Coast with tropical and subtropical lowlands with temperature of 25°C and high annual rainfall, the Central Andean where climate can range from temperate to subtropical with temperatures ranging from 8 to 22°C, and the Eastern Amazon with humid tropical forests and temperatures of 28°C and high annual rainfall (21). Moreover, Ecuador is the smallest of the Andean countries and is considered a megadiverse country with one of the largest concentrations of species worldwide, due to its unique environmental conditions that vary greatly depending on the region (22).

Inclusion Criteria:

1. Data from 2011 to 2021 (11 years) registered by the National Institute of Census and Statistics (INEC).
2. Cases diagnosed with the ICD-10 codes for filariasis (ICD: B74) in 221 Ecuadorian cities.
3. Hospital discharge diagnosis of:

- B740 (Filariasis due to *W. bancrofti*)
- B741 (Filariasis due to *B. malayi*)
- B742 (Filariasis due to *B. timori*)
- B743 (Loiasis)
- B744 (Mansonelliasis)
- B748 (Other filariases)
- B749 (Filariasis, unspecified)

Exclusion Criteria:

1. Cases that were not officially recorded by the National Institute of Census and Statistics (INEC).
2. Data from health centers that are not accredited in Ecuador.
3. Any diagnosis outside of the ICD-10 codes specified for filariasis.

Furthermore, it’s important to emphasize that our study is limited to only those cases that were officially recorded. Any other diagnosis that was not available or not covered under the ICD-10 codes for filariasis was excluded from our analysis.

### 2.3. Population

According to the 2017 National Institute of Statistics and Census (INEC) data projections, Ecuador has a population of 17,082,730, 51% women and 49% men; of which, around 9 million are in the coast, 7 million in the Andean region, about 900 thousand in the Amazon region and 30 thousand in the Galapagos Islands (23).

### 2.4. Outcome

The total number of cases reported during the period between 2011 and 2021, was analyzed. The information was integrated according to its distribution by geographic area provinces and cities (cantons), sex and age.

### 2.5. Data source and description

All cases of B740, B741, B742, B743, B744, B748, and B749 according to the 3-digit ICD-10 classification, were retrieved from INEC (20).

Continuous and categorical data was acquired at the national level. The following variables were analyzed: age; and sex. Where available, data was collated for the above diagnosed cases during a 11-year period, from the 24 provinces and the 223 cantons (political subdivisions of a province) in the country.

The overall incidence, mortality and case-fatality rate were calculated according to the entire population at risk and adjusted by sex and age for every province and canton as per the 2010 Census data and projections.

The hospital discharges and the annual mortality datasets are freely available as comma-separated values or D-Base database file format in the public INEC’s domain: http://www.ecuadorencifras.gob.ec/estadisticas-de-camas-y-egresos-hospitalarios-bases-de-datos/.

### 2.6. Data analysis

The variables analyzed in the study included sex, age, month, and year of hospital admission. Information regarding the type of medical care received (public versus private) and location was also included. Incidence rates were standardized by sex and age using projection data from canton and province based on the 2010 Ecuadorian census. Incidence rates were determined by dividing the number of new cases each year by the total population at risk for that year and for each age group. All cases were categorized into 17 age groups.

Moreover, adjusted incidence rates were computed by age, sex, and geographic location using stratified data for each group, following the given equation:


$$\text{Adjusted}\;\text{incidence}\;\text{rate}=\sum_{i=1}^{n}\left(\begin{array}{l} \text{Stratum}-\text{specific}\;\text{rat}e_{i}\\ \times \text{Standard}\;\text{population}\;\\ \;\text{proportio}n_{i} \end{array}\right)$$

Where:

$\text{Stratum}-\text{specific}\;\text{rat}e_{i}$: is the raw incidence rate for each age, sex, and geographic location stratum.

$\text{Standard}\;\text{population}\;\text{proportio}n_{i}$: is the proportion of the standard population that falls into stratum (*i*).

### 2.7. Ethical considerations

This analysis uses secondary data that is publicly available and anonymous. The data used cannot be linked to any personally identifiable information, as the data set did not include names, addresses, e-mail addresses, GPS locations or phone numbers. Since this secondary data is available on the official government website, no individual codes, or identification numbers were given, so it is impossible to link any data back to an individual.

## 3. Results

In Ecuador, a total of 26 hospital admissions and 3 deaths due to LF were recorded between 2011 and 2021. According to the official records, 23 cases (88.5%) were registered as Unspecified Filariasis, (B749), 2 cases (7.7%) were registered as *W. bancrofti* (B740) and only 1 case (3.8%) as Loaiasis (B743).

In terms of sex, men accounted for 62.5% (*n* = 17) of the total number of cases with an average incidence rate of 1.7 cases per every/1,000,000, while females accounted for 34.6% (*n* = 9), representing 1 case per every 1/1,000,000 woman.

### 3.1. Age and sex analysis

**Figure 2:**
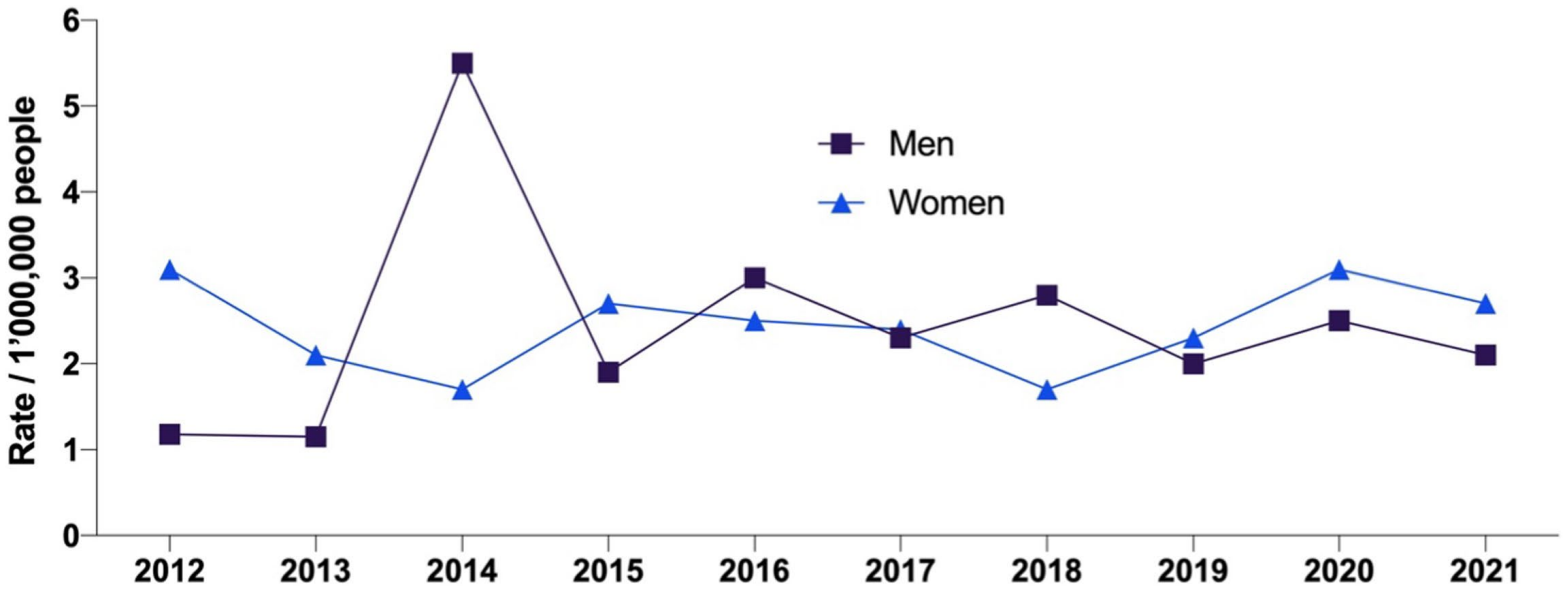
Lymphatic filariasis incidence rates from 2011 to 2021 by gender.

The average age of those patients who were hospitalized due to filaria was 45.18 years (SD: ± 18.13) for men and 50.11 years (SD: ± 17.62) for women. The temporal behavior of the incidence of LF was similar between men and women, however, in 2014 there was an increase in the number among men (Figure 2).

### 3.2. Incidence and mortality

The highest number of cases (*n* = 10) was found among patients aged 50–59 years. The overall sex-specific adjusted incidence rate was 2.44 per 1,000,000 cases for men and 2.43 per 1,000,000 for women (Table 1). The distribution of case and death rates varied significantly by age group. Although the population under 49 years accounted for 46.2% of cases, their mortality rate was 0%. In contrast, the highest mortality rate was observed in populations over 80 years of age (Table 1).

**Table 1:** Lymphatic filariasis incidence and mortality rates per 1,000,000 inhabits according to age and sex from 2011 to 2021.

	Women			Men		
Age	Cases (n)	Incidence rate / 1,000,000	Mortality rate / 1,000,000	Cases (n)	Incidence rate / 1,000,000	Mortality rate / 1,000,000
10–14	N/A	NA	N/A	1	1.2	NA
20–24	1	1.4	N/A	2	1.4	NA
30–34	1	1.7	N/A	2	1.6	NA
35–39	1	1.7	N/A	1	1.9	NA
40–44	N/A	N/A	N/A	1	2.0	NA
45–49	N//A	N/A	N/A	2	2.3	NA
50–54	2	2.4	N/A	3	2.8	NA
55–59	2	3.3	N/A	3	5.4	NA
60–64	1	3.2	N/A	N/A	N/A	NA
70–74	N/A	N/A	N/A	1	6.5	NA
>80	1	8.1	7.9	1	9.4	NA
**Total**	**9**	**2.4**	**7.9**	**17**	**2.4**	**3.7**

NA, not available data.

### 3.3. Geographic distribution

#### 3.3.1. Trends by province

LF incidence rates varied from province to province. Zamora Chinchipe had the highest rate (265 cases/1,000,000), followed by Santa Elena (61.7/1,000,000) and Esmeraldas (43.5/1,000,000). On the other hand, Manabí (12.5/1,000,000), Guayas (5.6/1,000.000) and Pichincha (5.2/1,000,000) reported the lowest rates (Table 2).

**Table 2:** Lymphatic filariasis incidence and mortality rates per 1,000,000 inhabits according to Ecuador provinces from 2011 to 2021.

Province	Cases (n)	Crude incidence rates / 1,000,000	Adjusted incidence rates / 1,000,000	Deaths (n)	Mortality rates / 1,000,000	Adjusted mortality rates / 1,000,000
El Oro	1	18.8	18.8	N/A	N/A	N/A
Esmeraldas	3	43.5	42.1	N/A	N/A	N/A
Guayas	2	5.6	5.8	2	6.8	6.8
Los Rios	5	24.0	24.6	N/A	N/A	N/A
Loja	N/A	N/A	N/A	1	86.8	86.8
Manabí	7	12.5	12.3	N/A	N/A	N/A
Pichincha	4	5.2	5.2	N/A	N/A	N/A
Santa Elena	1	61.7	61.7	N/A	N/A	N/A
Sto. Domingo DLT	1	32.9	32.9	N/A	N/A	N/A
Zamora Chinchipe	2	265.2	265.2	N/A	N/A	N/A

On the other hand, according to mortality rates due to LF the Ecuador’s provinces with the highest rates were Loja, and Guayas with 8.68, and 0.68, respectively (Table 2).

#### 3.3.2. Trends by canton

**Figure 3:**
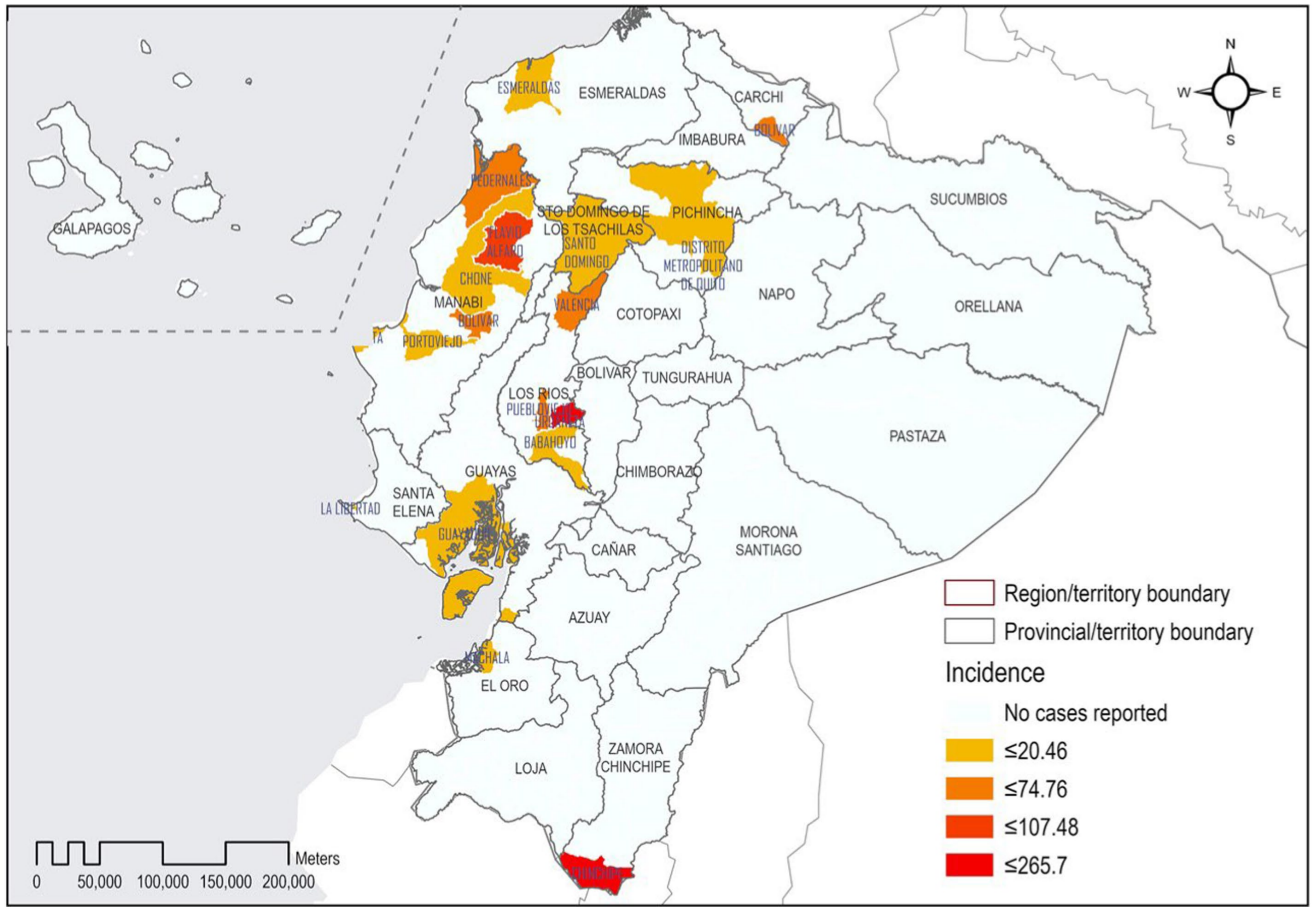
Geographic distribution of lymphatic filariasis in Ecuador by canton.

In Ecuador, 17 cantons have reported filariasis cases. From this small sample, the cantons with the highest LF incidence rates were Chinchipe with 265.67/1,000,000, followed by Urdaneta 210/1,000,000, and Flavio Alfaro with 107/1,000,000. On the other hand, Quito reported 6.0 cases/1,000,000 people followed by Guayaquil with 8.5/1,000,000 (Figure 3).

#### 3.3.3. Trends by type of establishment and sector

In analyzing the sources of filariasis reports across Ecuador, the data showcases a distribution among various types of medical establishments, both public and private. A predominant 67.9% (19 cases) of the filariasis reports emerged from establishments affiliated with the Ministry of Public Health. This was followed by 14.3% (4 cases) reported by the Ecuadorian Institute of Social Security. Additionally, the Ministry of Justice, Human Rights, and Worship and the Charitable Society of Guayaquil each contributed 3.6% (1 case) to the total count. On the private sector’s side, Private for-profit establishments documented 10.7% (3 cases) of the reports. It’s significant to highlight that out of these, only 3 patients received medical attention in the for-profit establishments, with 1 patient being seen in a not-for-profit private establishment.

### 3.4. Incidence rates by elevation analysis

The highest LF crude and adjusted incidence rate was found among cities located at lower altitudes. Using the low altitude range (<2,500 m), the adjusted incidence rate was 459/1,000,000 cases, while cities located above 2,500 m had a 98% lower incidence rate when adjusted by the population at risk (7.4/1,000,000).

## 4. Discussion

This study aims to elucidate the morbidity and mortality patterns as well as the spatial and altitudinal distribution of LF in Ecuador from 2011 to 2021. To the best of our knowledge, this investigation represents the inaugural study focused on LF within the Ecuadorian context.

We found a total of 26 cases of LF registered during the studied period, with a higher incidence in men. This gender difference is consistent with a review from 53 LF-endemic studies, indicating a lower prevalence in women (24). Several theories have been proposed to explain this phenomenon: the most widely accepted attributes it to a superior immune response and lower vector exposure in women (24). Yet, socio-cultural factors and gender inequality contribute significantly, as many women in LF-endemic countries are hesitant to report symptoms, particularly to male health workers. This reluctance affects their health status, with women having a 10-fold higher likelihood of developing elephantiasis in one leg than men (15, 25).

Regarding age, children under five are primarily asymptomatic, while symptomatic children face more severe outcomes from early lymphatic system damage. Adults in rural endemic areas are at the highest risk of vector-borne transmission (26, 27). Recent data indicate that young adults below the age of 20 have exhibited the highest rate of detected infections, a development attributable to advances in diagnostic technologies. These include a variety of immunological tests such as influenza assays (e.g., TropBio Og4C3 Test [TropBio, Australia]), immunochromatographic cards, experiential assays, and polymerase chain reaction tests (PCR) (18). Moreover, individuals aged between 15 and 44 have been identified as the demographic group with the highest infection rates. They constitute 58.5% of microfilaremic patients, 58.3% of those with hydrocele, and 47.2% of individuals affected by lymphedema (28).

Contrary to findings reported by several other authors, our study reveals that the age group most affected falls between 50 and 59 years old. In this context, the phenomenon of immunological “tolerance/intolerance” to the parasite appears to be a crucial factor during the initial exposure to the pathogen. If the infection is acquired during childhood, the initial lymphatic damage often goes unnoticed or is of mild or even low intensity (adenitis or adenopathy) and chronic lymphatic damage begins to appear during puberty, which is why there are more data in this age group. Subsequent research has indicated that the prevalence of *W. bancrofti* microfilaremia not only remains stable with increasing age but even shows a modest uptick, thereby confirming the phenomenon of immunotolerance (17). In women under the age of 20, clinical manifestations tend to be minor, peaking in severity around the age of 60. In men, such manifestations are most observed between the ages of 50 and 59 (24).

Although LF is highly debilitating and carries a substantial disease burden, it is infrequently fatal. Nonetheless, a large percentage of individuals afflicted with LF are susceptible to secondary bacterial infections, which, if untreated, can be life-threatening. Elevated rates of comorbidities such as obesity, hypertension, and diabetes have also been identified among patients with lymphedema (15). In our study, we documented 3 deaths due to LF, all of which occurred in individuals over 80 years of age. One plausible explanation for this may be the elevated risk this age group bears for contracting other diseases and succumbing to alternative causes of death.

Our findings suggest a greater burden in terms of years of life lost (YLL) among men as compared to women. The paucity of mortality data, combined with the rare fatal outcomes of LF, might result in an underestimation of the disease’s true burden (29). In a study by Ton et al., the mental health burden of LF on patients and their caregivers was assessed in terms of disability-adjusted life years (DALYs). The study posited that the YLL associated with LF were likely negligible (30). It is also noteworthy that these metrics may not adequately account for the influence of local contexts on the disease burden, particularly among impoverished populations (31).

Multiple vectors are responsible for the transmission of LF globally. In the Americas, the only documented vector is the female of the species *Culex quinquefasciatus* (16), which is prevalent in Ecuador, especially in regions with favorable environmental conditions such as the Ecuadorian Coast and the Amazon. A study conducted in the coastal region of northern Ecuador described the behavior of *C. quinquefasciatus* as likely being one of the most abundant mosquito’s species in the region, present in both urban and rural areas (32). These vectors have demonstrated the capability to travel vast distances, even across continents, involuntarily (33). Their presence has been reported in neighboring countries like Colombia and Peru, providing a possible explanation for their facile dissemination to Ecuador (34, 35).

In relation to the distribution by altitude, we observed that the majority of LF cases were concentrated in regions situated at elevations below 2,500 meters above sea level. This observation aligns with prior analyses concerning the global distribution of LF. Factors such as high rainfall, elevated temperatures, specific types of vegetation, and low altitudes have all been identified as contributing to an environment conducive for the survival of the mosquito vector, thereby increasing the likelihood of disease transmission (14). Nevertheless, the remarkable adaptability of this vector species allows for the possibility of its presence at varying altitudes. For instance, in the Central and Northern Sierra regions of Ecuador, we noted 5 cases at elevations exceeding 2,500 meters, which constitute 19.2% of the total observed cases. It is also worth mentioning that the upper altitudinal distribution limits for this vector have been reported to be 2,543 meters in Colombia and 2,327 meters in Venezuela (36). Alternatively, could be that patients caught it when they were in another region but were living in within the highlands when they were diagnosed.

Ecuador’s system of epidemiological surveillance is structured around both indicators and events, the latter relying on data collection methods that can be categorized as either active or passive. Moreover, the complexity of information analysis can range from simple to intricate. Among the health events prioritized for monitoring by the Ecuadorian government are neglected tropical diseases, subjected to active and complex surveillance. Despite this, our review of epidemiological data in Ecuador for the period under study revealed a conspicuous absence of LF reports within the country’s comprehensive epidemiological surveillance system. We hypothesize that this omission could be attributed to a procedural manual developed by the country’s highest health authority, which, paradoxically, does not yet include LF as of 2022 (37, 38).

Another potential explanation could be errors in the notification and registration of cases, typically handled by physicians working predominantly at the primary care level. This raises questions about whether the expertise of primary care providers in tropical diseases is sufficient and whether there are gaps in medical education in this domain. It is worth noting that one of Ecuador’s premier medical training institutions is in Quito, at an elevation of 2,800 m, where students are seldom exposed to clinical cases related to tropical infectious diseases, such as filariasis. Furthermore, we observed that a considerable proportion of registered cases (88.5%) were diagnosed as ‘filariasis, unspecified’ (B749). This may also indicate limitations within the healthcare system in making specific pathogen diagnoses. Given these multifaceted constraints, we conclude that the epidemiological notification process is suboptimal, leading to a significant underdiagnosis. As a result, we have a population suffering from a potentially debilitating disease that remains overlooked by health authorities.

Although Ecuador does not meet the criteria to be designated as a country endemic for LF, media outlets in recent years have sporadically reported cases of individuals afflicted with the disease. Unfortunately, these isolated reports have not been effective in galvanizing governmental authorities to implement appropriate control measures (39, 40). In light of this, our study aims to highlight the impact of this disease on the Ecuadorian population in recent years. It is our hope that these findings will serve as the requisite foundation for health authorities in Ecuador to institute control measures and provide support for those affected.

## 5. Limitations

The main limitation of this study is that to date Ecuador has not been reported as one of the endemic countries for LF, despite the cases reported, so the disease has been underdiagnosed. Moreover, this study is based on data obtained from the hospital admissions and discharges system, which only observed diagnosed cases. For this reason, it has not been possible to obtain all the information necessary to provide a more complete analysis of LF in Ecuador. Another limitation is the lack of studies of filariasis at the national level, since there are no data from previous studies, and as has been stated, the only reports come from non-scientific sources of information. However, as cases of LF with advanced progression usually require hospitalization, we consider that the number of cases found in this research may be very close to reality.

## 6. Conclusion

In epidemiological terms, this is the first report of its kind in Ecuador. Although there are limitations to the study, official data show that at least 26 cases of LF have been diagnosed in Ecuador. The incidence, although lower than in other countries, has resulted in at least three deaths being reported since 2011. Altitude is a factor associated with a lower predisposition to develop this type of pathology, which responds to the climatic variability of the vector.

## Data availability statement

The original contributions presented in the study are included in the article/supplementary material, further inquiries can be directed to the corresponding author.

## Author contributions

JI-C: Conceptualization, Data curation, Formal analysis, Investigation, Methodology, Project administration, Resources, Software, Visualization, Writing – original draft, Writing – review & editing. PN-L: Investigation, Methodology, Validation, Visualization, Writing – original draft, Writing – review & editing. JV-G: Investigation, Methodology, Software, Visualization, Writing – original draft. RF-N: Data curation, Investigation, Software, Validation, Visualization, Writing – original draft. RP-A: Investigation, Methodology, Resources, Validation, Visualization, Writing – original draft. MD: Formal analysis, Investigation, Resources, Validation, Visualization, Writing – original draft. SC: Investigation, Supervision, Validation, Visualization, Writing – review & editing. EO-P: Conceptualization, Funding acquisition, Investigation, Methodology, Project administration, Resources, Supervision, Validation, Visualization, Writing – review & editing.
